# A Comprehensive Exploration of Bioluminescence Systems, Mechanisms, and Advanced Assays for Versatile Applications

**DOI:** 10.1155/2024/8273237

**Published:** 2024-02-05

**Authors:** Asiri N. Dunuweera, Shashiprabha P. Dunuweera, K. Ranganathan

**Affiliations:** ^1^Department of Botany, University of Jaffna, Jaffna 40000, Sri Lanka; ^2^Department of Chemistry, University of Peradeniya, Peradeniya 20400, Sri Lanka

## Abstract

Bioluminescence has been a fascinating natural phenomenon of light emission from living creatures. It happens when the enzyme luciferase facilitates the oxidation of luciferin, resulting in the creation of an excited-state species that emits light. Although there are many bioluminescent systems, few have been identified. D-luciferin-dependent systems, coelenterazine-dependent systems, *Cypridina *luciferin-based systems, tetrapyrrole-based luciferins, bacterial bioluminescent systems, and fungal bioluminescent systems are natural bioluminescent systems. Since different bioluminescence systems, such as various combinations of luciferin-luciferase pair reactions, have different light emission wavelengths, they benefit industrial applications such as drug discovery, protein-protein interactions, in vivo imaging in small animals, and controlling neurons. Due to the expression of luciferase and easy permeation of luciferin into most cells and tissues, bioluminescence assays are applied nowadays with modern technologies in most cell and tissue types. It is a versatile technique in a variety of biomedical research. Furthermore, there are some investigated blue-sky research projects, such as bioluminescent plants and lamps. This review article is mainly based on the theory of diverse bioluminescence systems and their past, present, and future applications.

## 1. Introduction

As a result of a biochemical reaction, the emission of light by an organism is known as “bioluminescence,” which is a fascinating natural phenomenon of emitting light from living organisms. A bioluminescent system produces light through the oxygenation of a substrate, luciferin, and an enzyme, luciferase. Here, the luciferase enzyme catalyzed the production of an exciting intermediate molecule from oxygen that emits light when returning to the ground state [1]. Generating light in various bioluminescent systems often requires cofactors, supplementary enzymes, ATP, and intermediate steps. Regarding the timing of light emission, certain luciferases exhibit a distinctive mechanism: they bind to and stabilize oxygenated luciferin, emitting light only in the presence of Mg^2+^ or Ca^2+^ cations [2]. Bioluminescence stands apart from phosphorescence and fluorescence by not necessitating the preliminary absorption of sunlight or other forms of electromagnetic radiation by a molecule or pigment for the emission of light [3]. Hence, the surface quenching, surface scattering, and sample heating effects are hindered. Luciferin, owing to its nontoxic nature, bioavailability, high spatial resolution, sensitivity, and impressive quantum efficiency, finds applications in a range of analytical techniques within chemistry, biochemistry, and microbiology. These include both in vitro and in vivo methods such as immunoassays and bioimaging [4]. Nine natural luciferins have been resolved from more than 40 known bioluminescence systems, as shown in Table 1 [5]. Unfortunately, biochemical understanding of natural bioluminescent systems is limited, which hinders their widespread application.

## 2. Natural Bioluminescent Systems and Mechanisms

Bioluminescence has developed gradually in nature with distinct luciferases and luciferins. Especially, fireflies have evolved varieties of luciferases that belong to bioluminescent beetles. *Photinus pyralis* firefly luciferase (Fluc) was characterized with its substrate D-luciferin over half a century ago and is still utilized for a wide variety of research [6]. Furthermore, this can ligate CoA to fatty acids, the addition of catalyzing light emission from D-luciferin. The result is Fluc, a bifunctional enzyme that functions as both a long-chain fatty acyl-CoA synthetase (ACSL) and a luciferase [7]. Bioluminescence evolution has taken diverse paths in various organisms. For instance, the common D-luciferin substrate, oxidized by distinct luciferases in click beetles and fireflies, has evolved separately from acyl-CoA synthetase-like enzymes (ACSLs) [8]. Although marine luciferases catalyze light emission, these chemical structures are unrelated to luciferases, which have evolved to oxidize the marine luciferin coelenterazine. The biosynthesis of luciferin requires its specialized enzymatic pathways and several unique chemical transformations [9]. Benzoquinone is one of the intermediates in the biosynthesis of D-luciferin, and beetles utilize it for defense purposes. For example, bombardier beetles use benzoquinone and hydrogen peroxide to deter predators by creating a boiling-hot chemical spray [10]. In addition, attempts to identify luciferin in nonluminous species have proven unsuccessful. Some luciferins are formed nonenzymatically through the addition of cysteine to benzoquinone, indicating the possibility of in situ production of bioluminescent systems.

### 2.1. D-Luciferin-Dependent System

This system can be broadly seen in several lineages of beetles, including fireflies, click beetles, and railroad worms. The emitted light encompasses yellow, orange, and, in certain instances, red hues. These reactions involve D-luciferin, a stable and nontoxic compound. D-Luciferin is a protein with an approximate mass of 60 kDa and relies on ATP and Mg^2+^ for catalyzing the reaction [11]. Given that light emission intensity is contingent upon ATP concentration, this system finds extensive application in studies related to cancer metabolism and the analysis of water contaminated with bacteria. Numerous diagnoses rely on blood-related factors, which, in turn, are influenced by ATP concentration [12].

Essential enzymes in this system are as follows.

#### 2.1.1. Firefly Luciferase

**Figure 1:**
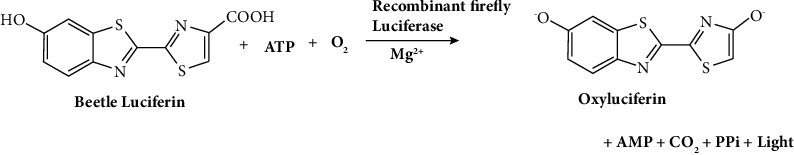
Chemical reaction of recombinant firefly luciferase with the substrate [15].

This is easy to produce in bacteria. It is available in thermostable mutant variants with enhanced spectral characteristics, yielding a high quantum efficiency of bioluminescence. [13]. Firefly luciferase has found extensive application in diverse in vitro and in vivo systems, facilitating the quantification of protein-protein and protein-ligand interactions, the assay of metabolites involved in cell communication and signaling, and the detection of pathogenic bacteria and viruses [14]. The chemical reaction of recombinant firefly luciferase with the substrate is given in Figure 1.

#### 2.1.2. Click Beetle Luciferases

This has been the second most popular D-luciferin-dependent luciferase group derived from *Pyrophorus plagiophthalamus* [16]. They emit light using four types of luciferases, which range from green (540 nm) to orange-red (593 nm) [17]. Due to their tolerance to a broad range of pH conditions, the existence of engineered variants, and the variability in color, these luciferases find extensive use in numerous applications.

### 2.2. Coelenterazine-Dependent Systems

This system was recognized as a substrate for bioluminescent reactions in luminous organisms spanning taxonomic distances and the most extensive diversity of bioluminescent organisms in marine ecosystems. Coelenterazine is produced from one phenylalanine and two tyrosine residues and is a modified tripeptide [18]. The emission range of all coelenterazine-dependent systems in nature is from 450 to 500 nm [14]. They do not necessitate any cofactors except for oxygen. In some cases, by a fluorescent protein, the color of bioluminescence is altered with the luciferase [19]. Among coelenterazine-dependent systems, the pH sensitivity, molecular weight, catalysis rates, and thermostability of luciferases vary. They are identified in *Renilla*, the decapods *Oplophorus*, the copepods, the scyphozoan medusa *Periphylla,* the fish *Benthosema pterotum*, the ostracods *Conchoecia*, and others [20]. These types of systems can be divided into photoprotein and luciferase types.

Several important luciferases utilizing coelenterazine or its analogs are as follows.

#### 2.2.1. *Renilla* Luciferase

**Figure 2:**
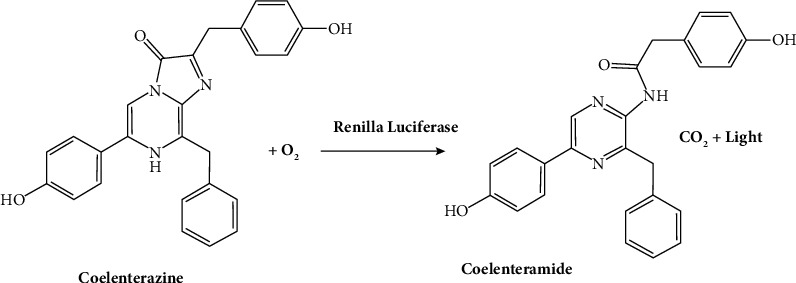
Chemical reaction of *Renilla* luciferase with the substrate [22].

It is a medium-sized cytosolic protein (36 kDa) derived from a coral, producing a steady luminescent signal [21]. This is commonly used in biomedical-related research, such as bioimaging and drug screening, due to the availability of engineered versions that offer enhanced brightness and red-shifted spectra. (Figure 2).

#### 2.2.2. *Gaussia* Luciferase

**Figure 3:**
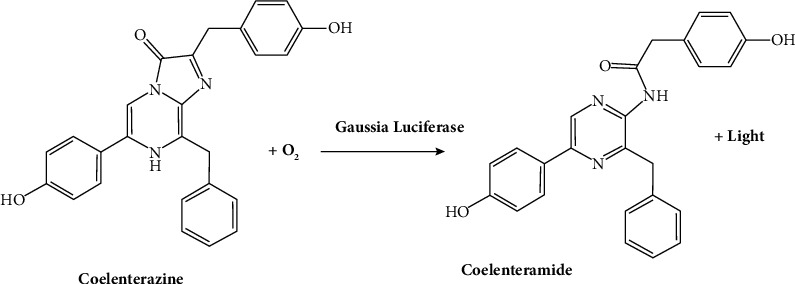
Chemical reaction of *Gaussia* luciferase with the substrate [24].

This small protein (20 kDa) is produced by a small crustacean of the Copepoda group [23]. This specific protein exhibits remarkable thermostability and a high catalytic rate. However, the alteration of its activity by forming disulfide bonds makes it unsuitable for certain heterologous systems (Figure 3**)**. This system is beneficial for monitoring tumor progression and drug response since the signals are linear with the number of cells [25].

#### 2.2.3. NanoLuc Luciferase

**Figure 4:**
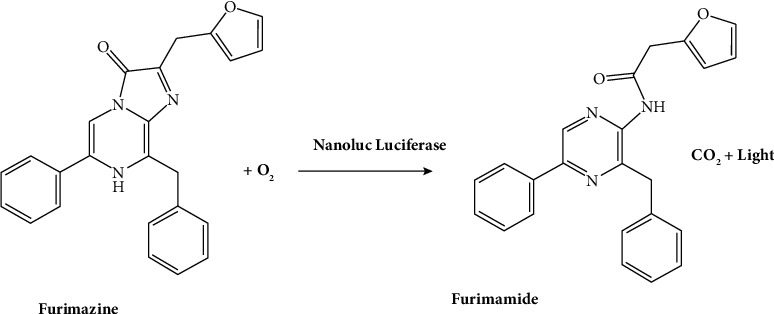
Chemical reaction of NanoLuc luciferase with the substrate [28].

This is from the shrimp *Oplophorus gracilirostris*, an engineered variant with a 19 kDa size, which produces a bright signal suitable for a broad range of applications [26]. This utilizes disulfide bonds and a cell-permeable coelenterazine analog [27]. These signals have been used for various applications (Figure 4). The drawback of this process lies in the cost of reagents. NanoLuc luciferase, in contrast, exhibits no indications of Post-translational modifications, disulfide bonds, or subcellular partitioning in mammalian cells. Unlike other luciferase variants, NanoLuc luciferase is exceptionally well-suited for both lytic and nonlytic reporter gene applications [29]. The diminutive size of the gene and its encoded protein proves advantageous for reporter virus applications and protein fusion. Furthermore, live-cell substrates are suitable for imaging applications.

### 2.3. *Cypridina *Luciferin-Based System

**Figure 5:**
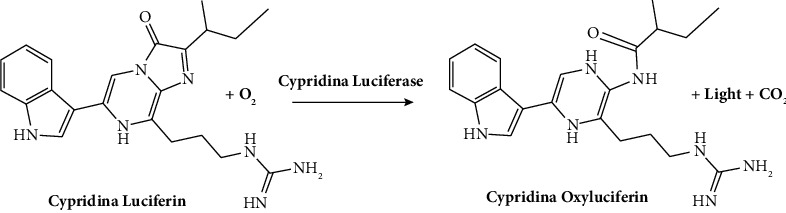
Chemical reaction of *Cypridina* luciferase with the substrate [30].

This is a blue light-emitting, and the respective metabolite is found in the ostracod *Cypridina* and *Porichthys*, a bioluminescent midshipman fish. This compound is produced from isoleucine, arginine, and tryptophan (Figure 5**)**. The *Cypridina* system finds extensive use in various applications, including studies of circadian rhythms, immunoassays, and bioimaging.

### 2.4. Tetrapyrrole-Based Luciferins

**Figure 6:**
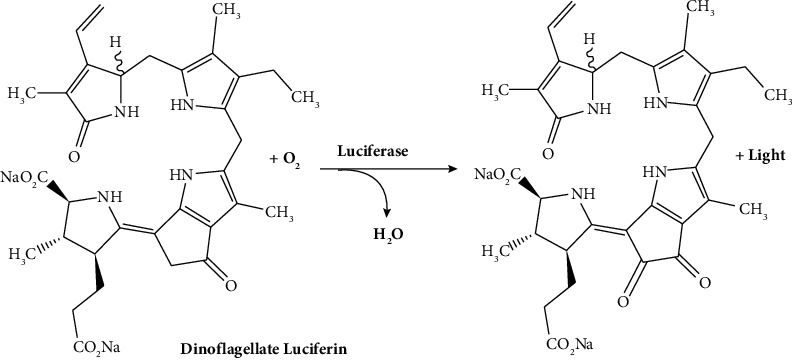
Chemical reaction of tetrapyrrole-based luciferase with the substrate [34].

Euphausiids (krill) and dinoflagellates (protists) form another large group of bioluminescent species by utilizing two very similar tetrapyrrole-based luciferins [31]. Bioluminescence occurs within distinct cellular structures known as scintillons in the dinoflagellates [32]. Light emission is induced through mechanical or electrical stimulation, making the organism visible to potential threats and attracting predators from higher trophic levels. This, in turn, serves as an effective defense mechanism. Euphausiids possess intricate organs equipped with specialized lenses for light emission [33]. These lenses can focus on light. These luciferases are very rarely used for research purposes (Figure 6**)**.

### 2.5. Bacterial Bioluminescent System

**Figure 7:**
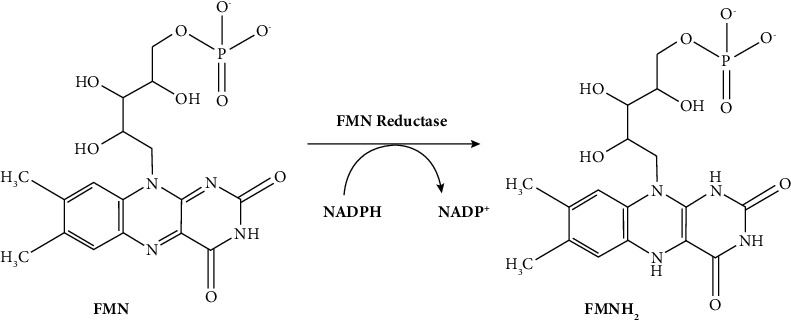
Chemical reaction of bacterial bioluminescent system [35].

For light emission, all bacteria utilize the exact and unique mechanism. In these reactions (Figure 7**)**, photons are produced by a set of reactions. It requires nicotinamide adenine dinucleotide (NADH), myristic aldehyde, oxygen, and flavin mononucleotide (FMN) [36]. The light sources of bacterial bioluminescence are FMN derivatives [37]. Nevertheless, these forms of bioluminescence, commonly referred to as luciferins, originate from the oxidation of myristic aldehyde. Bacterial luciferases assemble into a complex (75 kDa) comprising two polypeptide chains [38]. This complex is encoded by the lux operon and enzymes catalyzing luciferin biosynthesis.

Although red-shifted bioluminescence exists in both natural and engineered bacterial systems, it is generally distinguished by a blue hue, with a wavelength of around 490 nm [39]. The lux operon system stands as the only genetically encodable bioluminescent system. Overcoming challenges related to toxicity and inefficiency in eukaryotes proves to be a significant challenge in engineering autonomously glowing organisms across bacteria, yeasts, mammalian cell lines, plants, and beyond. Despite these findings, some studies exploit living bacteria as a light source, particularly in fields such as bacterial infections, environmental monitoring, and the creation of antimicrobial drugs. ILux represents the most luminous version of bacterial bioluminescence developed to date [40].

### 2.6. Fungal Bioluminescent System

**Figure 8:**
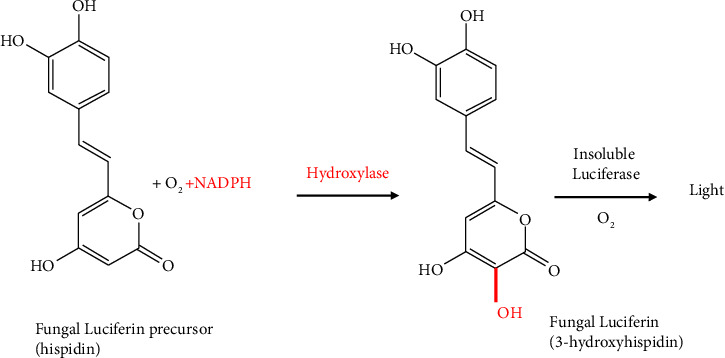
Chemical reaction of fungal bioluminescent system.

*α*-Pyrone 3-hydroxyhispidin is utilized by fungi, which is oxidized by an insoluble luciferase with required oxygen and results in the emission of green light (∼520 nm) [41, 42]. *Neonothopanus nambi* luciferase (nnLuz), in its wild-type form, demonstrates comparable performance to firefly luciferase and exhibits functionality across various heterologous systems (Figure 8**)** [42].

## 3. Modification of Genes for Applications

Modifications to genes are essential to activate bioluminescence, primarily because natural organisms may lack the specific genes responsible for generating the light-emitting proteins involved in this process. To incorporate bioluminescence into an organism, scientists commonly introduce or engineer genes encoding luciferase enzymes or other light-emitting proteins. In biotechnological applications such as to evaluate the transcriptional expression, luciferases from *Renilla*, deep-sea shrimp, bacteria, and beetles have been used [43]. However, the reporter gene activity should be optimized. The reporter gene should avoid anomalous expression, which only responds to effectors that the assay intends to monitor express uniformly and optimally in the host cells. It has low intrinsic stability to reflect transcriptional dynamics rapidly. Due to considerations in biology and enzymology, native luciferases are not as finely tuned as genetic reporters. As a result, extensive optimization has been undertaken to significantly minimize the risk of anomalous expression and enhance the destabilization of these reporters.

### 3.1. Peroxisomal Target Site Removal

Luciferase is located in specialized peroxisomes of photocytic cells in the light organs of fireflies [44]. The expression of firefly luciferase in foreign hosts can potentially disrupt normal cellular physiology and compromise the performance of the genetic reporter. This interference arises from the conserved translocation signal, a C-terminal tripeptide sequence (-Ser-Lys-Leu), which causes the accumulation of luciferase in glyoxysomes and peroxisomes [45].

Scientists have replaced the existing C-terminal sequence of the luciferase gene with -Ile-Ala-Val, a new C-terminal sequence, to develop an optimal cytoplasmic form [46]. This alteration leads to luminescence levels that are four to five times higher than those observed with the native enzyme expression in NIH/3T3 cells, a mouse cell line. *Renilla* and NanoLuc luciferase are not affected by peroxisomal targeting due to the absence of a targeting sequence [47].

### 3.2. Codon Optimization

Organisms exhibit a preference for specific codons over others due to the diverse encoding of amino acids. The protein translation efficiency can be increased in a nonnative host cell by adjusting the protein usage frequency when maintaining the same protein composition [48]. When considering the expression in mammalian cells, it is evident that the native luciferase genes from sea pansy (*Renilla reniformis*), deep-sea shrimp, and fireflies are suboptimal [49]. However, the expression level of luciferase can be increased significantly by altering the codons of the preferred ones. Nevertheless, it is crucial to simultaneously remove inappropriate or unintended transcription regulatory sequences when employed in mammalian cells.

### 3.3. Removal of Cryptic Regulatory Sequences

Anomalous reporter expression results from cryptic regulatory sequences in a reporter gene [50]. The cryptic regulatory sequence consists of a minimum of two transcription factor-binding sites separated by a spacer [51]. It can be either a promoter module or a transcription factor-binding site. Enhancer elements have the capability to elevate transcription levels in the absence of a promoter sequence or enhance the basal levels of gene expression in the presence of transcription regulatory sequences [52]. Those regulatory sequences can either increase or decrease the transcription process by affecting overall gene expression.

The luc2 gene has been created by removing the cryptic regulatory sequences in the Luc gene without changing the amino acid sequence [53]. In addition, wherever feasible, poly (A) addition sequences, *E. coli* promoters, or *E. coli* ribosome-binding sites, and sequences resembling splice sites have been removed [54]. The anomalous transcription can be avoided by reducing the number of cryptic regulatory sequences. This procedure can be applied to produce the hRluc gene by *Renilla* luciferase.

### 3.4. Addition of Degradation Signals

Reporter assays facilitate the quantification of the total accumulated reporter protein within cells, spanning the entire intracellular lifespan of the reporter. This measurement is affected by the stability of both mRNA and protein. As a result, the reporter offers a more accurate depiction of changes in gene expression, whether they involve upregulation or downregulation. Other than the firefly and *Renilla* luciferase reporter proteins, NanoLuc luciferase is more stable [55]. The responses from the reporter may experience a delay of several hours compared to changes in the underlying transcriptional events, mainly attributable to protein stability. To tackle this issue, scientists have developed destabilized luciferase reporters by genetically appending a protein degradation sequence to the luciferase reporter gene. This modification aims to improve the responsiveness of the reporter and reduce its cellular half-life. Two sequences, CL1 and PEST, are utilized to minimize the reporter half-life, both being protein degradation sequences [55]. The combination of these elements is referred to as rapid response genes, exhibiting a heightened reactivity to stimuli. Under moderate signal intensity, the luc2P gene demonstrates a swifter response compared to luc2, whereas the luc2CP gene exhibits rapid responsiveness with the least signal intensity [56]. These destabilized reporters give a response faster than nondestabilized reporters.

### 3.5. Secretion of Signal Addition

The generation of blue light occurs as the substrate coelenterazine undergoes oxidation facilitated by the inherent *Oplophorus* luciferase enzyme [57]. Shrimps release this enzyme as a defensive measure against predators. In laboratory settings, the secreted reporter proves advantageous for extracellular detection, circumventing cell lysis and enabling the exploration of cell viability. The secNluc gene has been engineered to optimize protein secretion efficiency in mammalian cells [58]. It is by replacing the native secretion signal with a mammalian protein secretion signal. Here, in the extracellular medium, the enzyme can accumulate.

### 3.6. Reporter Gene Vectors

Reporter vectors serve the purpose of transformation, the successful introduction of a gene of interest into a cell, and assessing the protein expression of that gene. These vectors contain a distinctive reporter gene. To investigate the transcriptional pathways, particularly those associated with regulatory regions, the expression of a reporter gene is used. Certain reporters also function as selective markers.

Reporter genes can be transferred into cultured cells by plasmids, which are extrachromosomal elements, especially plasmids [59]. Reporter vectors play a crucial role in the success of reporter assays. Instead of providing the reporter genes, these vectors offer promoters, polyadenylation sequences, multiple cloning sites, response elements, and other sequences. In addition, they include mammalian selectable markers to aid in the selection and expression processes [60].

#### 3.6.1. pGL4 Luciferase Reporter Vectors Encoding Firefly and *Renilla* Luciferase

**Figure 9:**
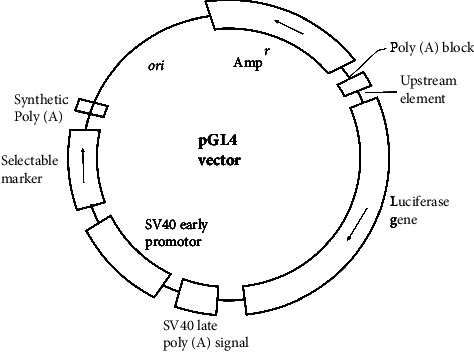
pGL4 luciferase reporter vector with additional features [64].

It causes anomalous responses and high background reading, for example, the mammalian reporter vector with the pGL3 luciferase reporter vectors [61]. Therefore, researchers have eliminated cryptic regulatory sequences whenever feasible while preserving reporter functionality. The multiple cloning sites have undergone redesign to facilitate the efficient transfer of desired DNA, incorporating the SfiI site [62]. Furthermore, a synthetic poly(A) site has been placed upstream of either the SV40 promoter or HSV-TK, CMV, or the multiple cloning region [63]. This results in the creation of a new unique vector called pGL4 (Figure 9).

#### 3.6.2. pNL Vectors Encoding NanoLuc Luciferase

Given that the pNL vectors are derived from the pGL4 vector backbone, they exhibit numerous similar advantages. These include a minimal promoter and viral promoters, minimizing the risk of anomalous results, such as cases with no promoter. In addition, they facilitate easy sequence transfer from existing plasmids [65]. The variations of the NanoLuc gene are optimized codons and removed restriction enzyme sites [66]. Certain coincident reporter vectors encode both firefly luciferases and NanoLuc within a single transcript. For the usage of high-throughput screening, these vectors do not have a promoter, CMV promoter, or minimal promoter [67].

#### 3.6.3. Bacterial Vectors

For consistent and homogenous expression levels, incorporating reporter genes into a bacterial genome is favored over episomal plasmid-based expression [68]. As this method does not necessitate antibiotic selection, alternative approaches to sustain episomal plasmids without antibiotic selection have been designed. For instance, *E. coli* strains that rely metabolically on diaminopimelic acid (DAP) have been established [69]. This allows for the preservation of plasmids containing DAP-producing genes, as plasmid loss results in bacterial cell death. Various methods, including temperature-sensitive vectors, transducing bacteriophages, bacteriophage integrase systems, and transposons, have been established to integrate genes into specific bacterial chromosomes. Numerous studies have documented the attenuation in strains expressing lux and GFP.

#### 3.6.4. Yeast Vectors

*Saccharomyces cerevisiae* cells are genetically modified to express the bioluminescent (BL) reporter gene luciferase from *Photinus pyralis* and the human androgen receptor (hAR), with regulation governed by the androgen response element (ARE) [70]. In the presence of androgens, the activated human androgen receptor (hAR) binds to the androgen response element (ARE) sequences, thereby initiating the expression of luciferase [71]. After introducing D-luciferin, luciferase activity measurements can be conducted, with the androgenic activity of the sample exhibiting proportionality to the bioluminescent signal (BL signal) [72]. A control yeast strain monitors the cytotoxicity of the sample, allowing BL signal correction according to cell viability.

## 4. Types of Bioluminescent Detection Assays

Exploring the fascinating realm of bioluminescence detection, various assays have been developed to unveil the secrets of light-emitting organisms. Utilizing bioluminescence as an assay tool gains enhanced potency due to its photon-emitting capability, broad compatibility with biological systems, and its natural occurrence in biological settings [14]. Bioluminescence chemistry can deliver 10- to 1,000-fold higher assay sensitivity than fluorescence assays [73]. Therefore, it can be applied to complex biological systems. In various applications (Table 2), the relationship between background and brightness affects the suitability of the assays. When photon detection is constrained, the background reading is influenced by the detection capabilities of instrumentation. In addition, in larger samples where photon detection is more efficient than optical requirements, the background reading becomes crucial. For instance, in experiments involving 96- and 384-multiwell plates or image analysis in mice samples, a lower background reading enhances sensitivity. In bioluminescence assays, efficient photon detection is achieved as photodetectors can be placed in close proximity to the samples, eliminating the need for monochromators or optical filters [78]. Therefore, bioluminescence is measured approximately at 10–20 moles less than 10,000 molecules per sample [79]. For a typical biological sample, these are a few molecules per cell. Many bioluminescent assays are linear to 6–8 log values of the analyte concentration [80]. Nevertheless, luminometers can function across these ranges. With the exception of the reporter gene assay, the concentrations of all components remain constant. In reporter gene assays, the variable component is the enzyme, and either luciferin or ATP may serve as the variable component [81].

### 4.1. Reporter Gene Activity Measuring

Bioluminescent detection assays rely on controlling and harnessing the light-emitting chemistry of the enzyme, utilizing its substrate at analytical levels. If one component remains constant while the other components vary in concentration, the emitted light is directly proportional to the variable component [82]. This is a measurable parameter where the variable component can be a cofactor, substrate, or the luciferase enzyme itself. Quantification of intracellular luciferase can be achieved by introducing the luciferase substrate to initiate the luminescent reaction or a buffered solution containing detergent to lyse the cells [83]. The signals reduce slowly with time due to the decaying of luminescence. Typically, reporter gene assays involve either one or two reporter genes [84]. To standardize the outcomes of the experimental reporter, a second reporter is expressed from a “control” vector. The control reporter gene is regulated by a constitutive promoter. Various reporter genes are employed to assay the products individually. Alternatively, dual-reporter assays can be devised by incorporating both reporter genes, offering the advantage of efficiently extracting more information in a single experiment [85].

### 4.2. Single-Reporter Assays

These assays contain a single reporter and give quick results for gene expression data from the cells [86]. If the information from a single reporter is insufficient for accuracy, it is better to move to the dual-reporter assays. Since the bright reactions fade quickly, consideration of luminescence intensity and duration is essential.

### 4.3. Dual-Reporter Assays

These assays employ two reporters, both of which are luciferases. As both firefly and *Renilla* luciferase activities are utilized in these assays, NanoLuc and firefly luciferases can also be employed [87]. These two luciferases can be differentiated by the specificities of the enzymes, and they use different substrates. Due to the higher sensitivity of NanoLuc luminescence and better quenching of firefly luciferase activity, this reporter combination has several advantages such as lower background levels [88]. Furthermore, both *Renilla* luciferase and firefly systems provide a longer luciferase signal half-life [89]. Enhancing experimental accuracy and efficiency can be achieved through dual-reporter assays. Hence, it becomes imperative to normalize potential interfering factors inherent in the experimental system, equalize variations in transfection efficiencies among samples, and minimize variability that could obscure significant correlations [90].

### 4.4. Multicolor Luciferase Reporter Assay

Although the dual luciferase assay is possible for intracellular detection of bioactive compounds *in vitro* HTS (high-throughput screening) and simultaneous monitoring of gene expression, it requires many samples [91]. Therefore, it is easy to have an improved and developed system to simultaneously evaluate gene expression, ligand binding, protein-protein interaction, and posttranscriptional modifications in a one-step reaction in single-cell extracts. It facilitates the interpretation of data since it requires only reduced samples, and it saves money. The reaction of D-luciferin and beetle luciferases produces distinct colors, easily separated by an optical filter [92]. CBRluc, CBGluc, SLR, ELuc, SLO, and SLG are beetle luciferases that display a wide spectral range (Table 3) [103]. In this context, three reporter plasmid vectors have been crafted, each featuring distinct promoter sites labeled as Promoter 1, Promoter 2, and Control Promoter. These vectors encompass the beetle luciferase gene sequence, emitting three distinct colors: green, orange, and red. Following the transfection of the mentioned plasmids, the expression of three different luciferase genes is regulated by the respective promoter regions. The resulting green, orange, and red-emitting luciferase proteins catalyze the reaction with firefly luciferin to produce light in their respective colors [104]. The luciferase-expressing cells are lysed in some assays, such as transient transfection multicolor luciferase assays [105]. Subsequently, the quantities of expressed luciferase proteins are determined based on their respective light intensities. This outcome encompasses the levels of control promoter activity and two distinct target promoter activities within living cells. During the data analysis phase, the control promoter activity (red luciferase) is normalized against the two target promoter activities (green and orange, corresponding to Promoter 1 and Promoter 2, respectively) [87]. In this system, green-emitting luciferase, orange-emitting promoter, and red-emitting promoters are used from *Rhagophthalmus ohbai* (SLG; *λ*_max_ = 550 nm), a point mutant of the green-emitting luciferase (SLO; *λ*_max_ = 580 nm), and *Phrixothrix hirtus* (SLR; *λ*_max_ = 630 nm), respectively [106].

Due to emitting light of all the abovementioned luciferases with firefly D-luciferin, the activities are detected in a one-step reaction in a single sample [85]. For that, different filters can be used with >560 nm (O56) and >600 nm (R60) long-pass filters. First, without any filter, the total relative light units (RLUs) are measured. Then, RLU values through O56 and R60 are measured separately [107]. Each luciferase activity is calculated by the following formula:

(1) $$\begin{array}{c} \left[\begin{array}{c} F_{0}\\ F_{1}\\ F_{2} \end{array}\right]=\left[\begin{array}{ccc} 1 & 1 & 1\\ {\kappa \text{G}}_{O56} & {\kappa \text{O}}_{O56} & {\kappa \text{R}}_{O56}\\ {\kappa \text{G}}_{R60} & {\kappa \text{O}}_{R60} & {\kappa \text{R}}_{R60} \end{array}\right]\left[\begin{array}{c} G\\ O\\ R \end{array}\right]. \end{array}$$

Here, *F*_0_ = total RLU, *F*_1_ = RLU through O56, and *F*_2_ = RLU through *R*60, *G*, *O*, and *R* = the activities of the green-, orange-, and red-emitting luciferases, respectively, *κ*G_O56_, *κ*O_O56_, and *κ*R_O56_ = the transmission coefficients of the green-, orange-, and red-emitting luciferases of the O56 filter, respectively, and *κ*G_R60_, *κ*O_R60_, and *κ*R_R60_ = transmission coefficients of the green-, orange-, and red-emitting luciferases of the R60 filter, respectively [107].

### 4.5. Real-Time Luciferase Reporter Assay

Upon introducing a bioluminescent substrate into cells, the resulting luminescence can be longitudinally and quantitatively measured with real-time resolution in both in vitro and in vivo systems [108]. This is one of the advantages of luciferase reporters. The advantages of employing the firefly luciferase and firefly D-luciferin pair for potential luciferase-luciferin reactions include accurate quantification of generated luminescence. Firefly D-luciferin readily permeates cells and tissues, demonstrating high stability, and contributes to negligible background values [109].

In this assay, the reporter plasmid vector incorporates both the luciferase gene and target promoter sequences. Upon introducing the plasmid into the designated living cells, the promoter region governs the expression of the luciferase gene. In a real-time luciferase assay, luciferin present in the medium permeates the living cells [110]. The luciferase expressed in cells oxidizes luciferin immediately to produce light, which can be measured by using a luminometer [43]. ATP is a prerequisite for this reaction. The intensity of light is directly linked to the promoter's activity in living cells.

### 4.6. NanoBiT system

This system is based on two small subunits, small BiT (SmBiT) and large BiT (LgBiT) of the very bright NanoLuc luciferase [111]. The individual optimization of these subunits ensures minimal self-association and enhanced stability. These subunits are expressed as fusions with target proteins of interest. The interaction of the target proteins promotes subunit complementation, leading to the generation of a bright, luminescent enzyme [111]. Protein-protein interaction dynamics can be followed in real-time inside living cells using a nonlytic detection reagent that contains an optimized cell-permeable substrate.

## 5. Application of Bioluminescence in Research

Bioluminescence reporters find application in diverse biological functions, including protein-protein interaction, gene expression, and posttranslational modification within cell-based assays. The utilization of bioluminescence has made substantial contributions to the field of biomedical research (Table 4). This technology provides great interest in monitoring the different biological processes in neuroscience, virology, immunology, and oncology.

### 5.1. Reporter Gene Applications

Reporter genes are usually used to analyze and dissect the function of cis-acting genetic elements and analyze gene expression and regulation. The fundamental principle involves attaching a reporter gene, often a gene encoding a readily measurable protein or enzyme, to the gene of interest. Changes in the expression of the target gene are reflected in the activity of the reporter gene product, simplifying the detection and quantification of gene expression levels. This approach has widespread applications, ranging from elucidating promoter activity and transcriptional regulation to assessing the impact of various factors on gene expression dynamics. Reporter gene assays are pivotal in unraveling the intricacies of cellular processes, aiding drug discovery, and developing novel therapeutic strategies. However, they can be used to study other cellular events, including examining cell health and signaling pathways unrelated to gene expression [85].

#### 5.1.1. Normalized for Changes in Cell Physiology

Certain activities linked to cell physiology may influence the expression of the reporter gene. Nevertheless, employing multiplexed assays allows for the examination of correlations within cells. For example, the coupling of target suppression by RNAi can be assessed for its impact on cellular physiology [128]. Inducing degradation of target mRNA, a gene can be specifically deactivated by double-stranded RNA that is complementary to target mRNA [129]. This phenomenon is recognized as RNA interference (RNAi). Consequently, RNAi, or more broadly, RNA silencing, has become a potent tool for analyzing gene function [130]. There is a decrease in firefly luciferase expression, when introducing miRNAs or binding endogenous to the cloned miRNA target sequence.

### 5.2. Cell Signaling Pathway Assessing

Luciferase reporter assays find extensive use in exploring cellular signaling pathways and serve as a screening tool in drug discovery. To pinpoint signaling pathways and monitor signal transduction, synthetic constructs containing cloned regulatory elements directing reporter gene expression are employed [131]. In order to investigate the factors implicated in the response, inhibitors and siRNAs can be utilized. Multiple firefly luciferase pGL4 vectors have been designed based on regulatory sequences and various response elements to characterize and modulate signaling pathways [132].

### 5.3. Nuclear Receptor Examination

**Figure 10:**
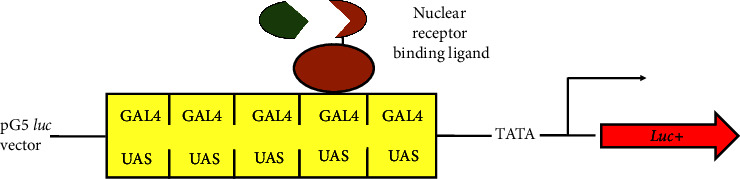
The nuclear reporter assay.

Bioluminescent reporter genes, such as nuclear receptors, have been used to investigate the presence of steroids and other molecules within the cell. Nuclear receptors are typically located in the cytoplasm and often form complexes with associated regulatory proteins [133]. Translocation into the nucleus is initiated by the binding of the ligand, resulting in the upregulation of the neighboring gene. (Figure 10). Bioluminescent reporters can be identified and characterized as corepressors, coactivators, nuclear receptor agonists, and antagonists by a universal receptor assay [134]. Furthermore, in cellular and animal models, light-emitting reporters' luciferase reporter genes have been used.

### 5.4. Bioluminescence-Induced Photouncaging of Small Molecules

The BRET-induced reaction can be applied to release small-molecule drugs like ibrutinib, esomeprazole, rosuvastatin, and duocarmycin. Lindberg et al. reported the first demonstration of a BRET-based chemoselective bioorthogonal chemical reaction. Energy transfer took place from a bioluminescent protein (NanoLuc luciferase) to a ruthenium-based photocatalyst, leading to the uncaging of the substrate. A novel template named LUPIN was introduced for conjugating the photocatalyst with nucleic acids. LUPIN served as a flexible platform, functioning effectively at low concentrations (2–50 nM), and resulted in the uncaging of the catalytic substrate, yielding 100–600 nM product [135].

### 5.5. Bioluminescence-Based Photodynamic Therapy (PDT)

Photodynamic therapy (PDT) is a phototherapeutic technique that employs light and a photosensitizer in conjunction with molecular tissue oxygen to induce cell death. The incident energy from the light source needs to be suitable for exciting the photosensitizer, leading to the generation of reactive oxygen species. Currently, only a limited number of other BRET-based PDT agents have been documented. A new biocompatible, multifunctional, and self-illuminating PDT agent consisting of a fused protein (Gaussia luciferase-Listeria innocua DNA-binding protein from starved cells) conjugated with zinc (II) protoporphyrin IX was introduced by Al-Ani et al. The PDT agent was effectively delivered to tumor cells and suppressed the proliferation of breast cancer cells through the generation of ROS [136].

### 5.6. Bioluminescence Imaging

Bioluminescence imaging is an economical and noninvasive technology designed for the real-time examination of biological processes at the molecular level. D-Luciferin serves as the substrate for the bioluminescence reaction catalyzed by firefly luciferase. The reaction produces a broad-band visible light emission spectrum within the range of 530–640 nm. In imaging, specialized supercooled charge-coupled device (CCD) cameras detect emission photons that have penetrated through tissues. The sensitivity of photon detection relies on factors such as the tissue's penetration depth, luciferase expression intensity, and the sensitivity of the detection system [137].

In contrast to traditional fluorescence imaging, bioluminescence imaging in vivo exhibits no autofluorescence in biological tissues. While most in vivo bioluminescence imaging has historically been confined to the visible region, there has been recent development in the creation of bioluminescence probes that comprise red-shifted bioluminescent protein-substrate systems, aiming to exploit the near-infrared (NIR) region [138]. To implement in vivo bioluminescence, luciferase-expressing cells, including tumor cells, immune cells, and stem cells, can be considered. In this method, a luciferase-expressed stable cell line should first be obtained by gene transfection. Then, these cells are injected into a biological system for imaging. An alternative approach is the creation of a luciferase protein probe. Following fusion with a specific antibody, luciferin is administered, initiating the bioluminescence imaging process within the system [139]. Morciano et al. studied a comprehensive protocol to measure the ATP concentration in biological systems using targeted luciferase probes [140].

### 5.7. Bioluminescence Resonance Energy Transfer (BRET)

BRET proves advantageous for investigating protein-protein interactions within cells and has been employed as an alternative approach to achieve in vivo bioluminescence imaging at extended wavelengths [109]. The theoretical foundation of BRET relies on Forster resonance energy transfer, achieved through nonradiative dipole-dipole coupling from a bioluminescent donor (a luciferase-luciferin system) to a fluorescent acceptor. As per the established equation for BRET (equation (2)), the efficiency of this process is inversely proportional to the sixth power of the distance between the bioluminescent donor and the fluorescent acceptor:

(2) $$\begin{array}{c} E=\frac{1}{{1+\left[r/\left(0.21{\left[\kappa^{2}Q_{D}n^{-4}J\left(\lambda \right)\right]}^{\left(1/6\right)}\right)\right]}^{6}}, \end{array}$$

where *r* is the distance between the bioluminescence donor and fluorescent acceptor, *Ro* is the Förster distance at which the BRET efficiency is 50% (equation (3)), *κ* is an orientation parameter,

(3) $$\begin{array}{c} R_{0}={0.21\left[\kappa^{2}Q_{D}n^{-4}J\left(\lambda \right)\right]}^{\left(1/6\right)}. \end{array}$$

*Q*_*D* _ is the quantum yield of the bioluminescence donor, *n* is the refractive index of the cellular medium, and *J*(*λ*) is the spectral overlap integral between the normalized donor emission and the acceptor excitation spectra [141]. Tsuboi et al. established the application of BRET-based dual-color imaging (visible green/near-infrared) for membrane receptors in tumor cells. This was achieved using a quantum dot (Q_D_) and luciferase protein conjugate [142]. FRET-based probes for caspase activity have been employed to observe apoptotic cell signaling. Nevertheless, a major limitation arises from autofluorescence and light scattering induced by the external energy source. Therefore, Hamer et al. have developed BRET sensor proteins to measure intracellular caspase activity, offering a potential solution to these challenges [143].

### 5.8. Bioluminescent Biosensors

Bioluminescent sensing is employed to identify alterations in luminescence emitted by bacteria in reaction to a specific target analyte. As a result, bioluminescent biosensors serve as a powerful tool for the detection of bacterial bioluminescence. For example, the oxidation of flavin mononucleotide (FMNH_2_) and a long-chain fatty aldehyde induces the emission of blue-green light [39]. This process is facilitated by luciferase, encoded by the lux gene. As it measures enzyme activity rather than protein quantification, this method ensures faster and more sensitive detection than fluorescence. Notably, these bioluminescence reactions do not necessitate external substances, as all reactants are generated in vivo through the lux genes [144]. This method has proven effective in identifying toxicants, even at extremely low concentrations. Furthermore, bioluminescent sensing has been successfully applied for the detection and monitoring of contaminants in soil, water, and air [145]. Furthermore, these sensors provide a specific, cost-effective, and reliable assessment method for evaluating various contaminants and have been applied to detect toxicants in food at minor levels and monitor bacterial contamination and count in food.

## 6. Blue Skies Research for a Sustainable Future

Blue skies research in bioluminescence represents an exciting frontier in scientific exploration, driven by the pursuit of understanding the fascinating phenomenon of living organisms emitting light. Unlike directed research aimed at immediate applications, this inquiry delves into the mysteries of bioluminescence with a spirit of curiosity and discovery. Bioluminescence, a natural occurrence in certain organisms, holds immense untapped potential for a sustainable future. By unraveling the underlying mechanisms and functions of bioluminescence, scientists aim to not only illuminate the intricate workings of these organisms but also harness this knowledge for innovative applications in diverse fields such as medical diagnostics, environmental monitoring, and beyond. This blue skies research opens doors to unexpected possibilities, paving the way for sustainable solutions inspired by nature's own illuminating phenomena.

### 6.1. Bioluminescent Lamps

There have been several projects on introducing bioluminescent dinoflagellate lamps to decorate houses, roads, and buildings. Furthermore, bioluminescent bacteria have also been used to develop lamps as a form of sustainable lighting. For that, we should increase the lifespan of bacteria by genetically lighting the lamps for more days.

### 6.2. Glowing Plants

Plants, characterized by chlorophyll instead of hemoglobin, possess a distinct optical window compared to mammals. Consequently, the utilization of bioluminescence imaging in plants is less prevalent. Chlorophylls in plants absorb light at two distinct wavelengths: *λ* = 450 nm and *λ* = 650 nm [146]. Due to the lower circulation of water and nutrients in plant cells than in mammalian cells, there is a challenge to transfer luciferin in cells. As a result, the transfer of bioluminescence images is constrained to cell cultures or seedlings cultivated in Petri dishes within laboratory settings. To address this limitation, a genetically encoded Fluc reporter gene was incorporated into a gene controlled by the promoter of the chlorophyll a-b binding protein (Cab2). Cab2 is a membrane protein that plays a crucial role in photosynthesis [147]. *Arabidopsis thaliana,* a versatile model organism used in the biology laboratory, has been grown on sterile cell culture plates by spraying a luciferin solution [148]. Bioluminescence imaging has been employed to visualize the organ localization of Cab2 under both dark and light conditions. Addressing challenges in delivering D-luciferin to the cells of interest, there is a proposal to explore the development of genetically modified plants [3]. Consequently, genetically modified plants were created at the conclusion of this experiment. In these plants, the bacterial lux operon was expressed in plastids to generate light. However, the system faced challenges as the strong absorption of blue light by chlorophyll resulted in insufficient light emission, and it was observed to be toxic to the plants [149]. Hence, there has been a debate about creating genetically engineered plants with fungal genes to make them autoluminescent. Unlike laboratory-based plants, these organisms could ideally grow in soil. The vision is for these plants to evolve into green light-emitting plants in the future.

### 6.3. Arts

Due to the wide spreading of bioluminescence systems, artists also look towards innovations and novel applications in living arts and novel arts. Bioluminescence from single-celled dinoflagellates and bioluminescent bacteria is widely used. They are based on the Petri dishes, emitting light when growing, and finally, they fade the light when dying. This almost takes one to two weeks.

### 6.4. Tester for Water Purity

Genetically modified bioluminescent microorganisms have been engineered to grow in challenging conditions, allowing them to grow and emit light in polluted water, facilitating effective identification [150]. Hence, these microorganisms can be additionally engineered to luminesce and react to specific toxins, providing a straightforward means to assess the toxicity of water. This approach is applicable for evaluating substances such as oil hydrocarbons and arsenic, common contaminants in water.

### 6.5. Medical Tools

Utilizing bioluminescent molecules for cell tracking enables rapid identification of infectious agents, the localization of cancer cells, and immune system response cells. Moreover, this approach can be employed to study live tissues [151]. The GFP gene has been integrated into the genetic code of various species, enabling them to produce their own GFP and related proteins [152]. Similar approaches can be employed by introducing the GFP gene into the genomes of birds, plants, nematodes, and fish species. Likewise, to investigate some neurological diseases, such as Parkinson's, using rodent models is not accurate. Therefore, some research is underway to insert bioluminescence genes into the mammalian genome to create an animal model. For example, GFP gene-carrying viruses were injected into marmoset monkey embryos, and bioluminescent babies were born; to investigate HIV transmission, FIV, which moves the similar vector of HIV, has been injected to unfertilized cat eggs [153]. Factor Xa is a blended bioluminescent agent that consists of firefly luciferase and a unique dye [154]. It can be applied to assess the efficacy of heparin treatment with minimal amounts of blood protein. For monitoring the transmission of HIV between heterosexual males and females, luciferase-inserted SIV (simian immunodeficiency virus), exhibiting similar behavior to HIV, has been employed [155]. Bioluminescent imaging has been employed to comprehend the transport functions of the blood-placenta barrier (BPB), and analogous technology can be applied to comprehend the equivalent function of the blood-brain barrier [156]. Researchers are hopeful to apply this translation to human studies soon.

## 7. Limitations

There are challenges associated with using luciferase-based products and conducting experiments based on bioluminescence imaging. In luciferase-based in vitro experiments, reliance on relative luminescence units (RLUs), which are arbitrary units, makes standardizing in vitro bioluminescence assays a difficult task. Furthermore, RLU depends on the luminometer or photon detector. High sensitivity bioluminescence imaging depends on the background and media; background control is essential. For example, for *in vitro* assays using cell lysates or supernatants, the same buffer or saline solution is necessary for background reading. Some autoluminescence substrates such as coelenterazine also emit light without enzymes and increase the background readings. Furthermore, some extracellular and intracellular factors also directly affect the readings, for example, H_2_O_2_, temperature, pH, and proteolytic degradation of enzyme.

The enzyme stability also dramatically affects the outcome. Thus, checking the enzyme half-life and exogenous and endogenous factors affecting the enzyme activity is essential before the experiments.

As oxygen serves as the limiting factor in all luciferase reactions, conducting bioluminescence assays becomes impractical under anaerobic conditions and in hypoxic tissues, such as tumor cells. The substrate concentration also affects the RLU. Therefore, the light emission is directly proportional to the luciferase concentration. To generate strong photon emission in *in vivo* assays, a sufficient substrate amount should be reached in the luciferase-expressing cells. Adequate intake of the substrate should be found in nonluciferase-secreting cells. Therefore, it depends on the membrane permeability to the substrate and the location of the luciferase-expressing cells. Some pigmented molecules such as hemoglobin and melanin absorb light, and some highly vascularized organs emit lower light intensities than lower vascularized organs.

## 8. Conclusion

In summary, bioluminescence, recognized as “cold light,” where less than 20% of emitted light produces heat, is primarily orchestrated by marine organisms. Some bioluminescent entities, incapable of synthesizing luciferin, establish symbiotic relationships to acquire it, while others independently synthesize luciferin. The by-product, oxyluciferin, results from the interaction of oxygenated luciferin and luciferase, generating light as a consequential outcome. Genetic engineering, a focal point of bioluminescence research, aims to enhance life's ease and safety. We have discussed natural bioluminescent systems such as various D-luciferin-dependent systems, coelenterazine-dependent systems, *Cypridina *luciferin-based system, tetrapyrrole-based luciferins system, bacterial, fungal bioluminescent systems, and their mechanisms. Nluc/coelenterazine system is an engineered system that is not commercially available, although it has a higher photon output. Furthermore, some luciferin systems are not used much due to their poor availability commercially. Reporter genes are designed to attach other genes to function. Developing more complementary luciferin and luciferase pairs with improved properties with more novel applications is necessary. Therefore, different gene modifications and the assays used for the bioluminescence application were explained in detail. Bioluminescence imaging is a more sensitive, novel technology to investigate cell physiology than fluorescent imaging techniques, which use fluorescent proteins such as green fluorescent protein (GFP) and its derivatives. Advanced luciferase technologies play a pivotal role in quantitatively visualizing gene expression at the intricacies of single-cell resolution. Researchers use sensitive charged-coupled device (CCD) cameras to capture real-time images of the luminescent processes. This marks a significant leap in our understanding and utilization of bioluminescence across various historical and contemporary research applications. A meticulous analysis of future prospects and limitations in bioluminescence imaging has been undertaken, laying a robust foundation for upcoming scientists to navigate and explore the vast potential embedded in this illuminating field.

## Figures and Tables

**Table 1:** Molecular structures of the known nine natural luciferins.

Luciferin name	Discovery	Luciferin structure
D-Luciferin	McElroy 1957	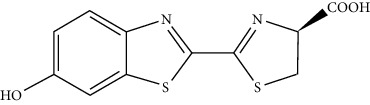

*Cypridina* luciferin	Shimomura 1957	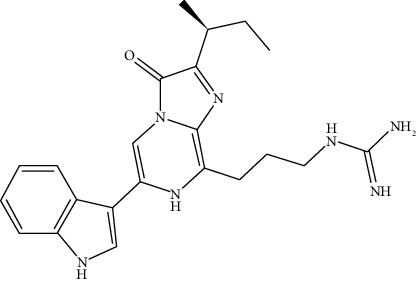

Bacterial luciferin	Cormier 1963	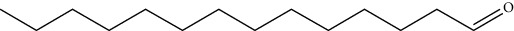

Latia luciferin	Shimomura 1968	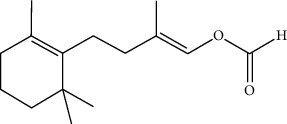

Coelenterazine	Inoue 1976	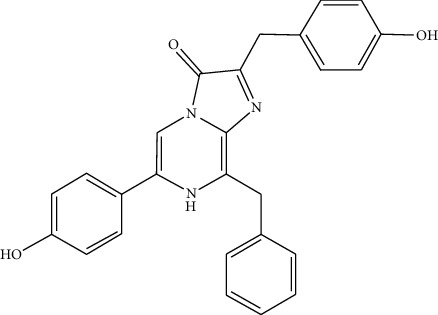

*Diplocardia* luciferin	Ohtsuka 1976	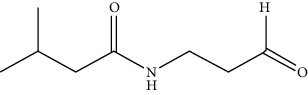

X = OH rill luciferin	Nakamura 1989	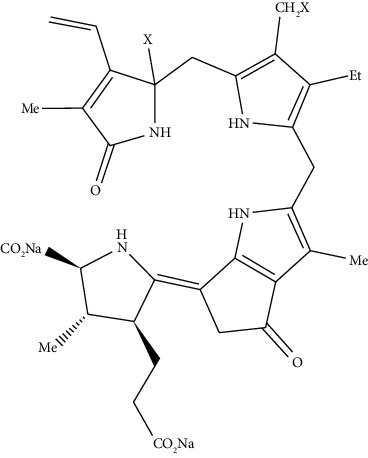
X = H dinoflagellate luciferin		

*Fridericia heliota* luciferin	Petushkov, Yampolsky 2014	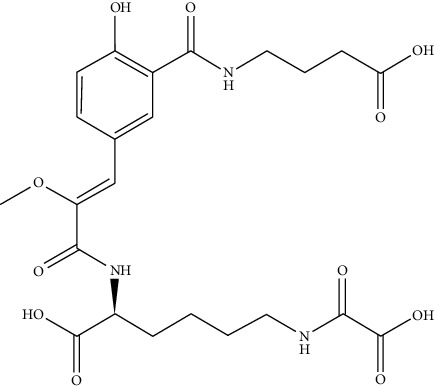

Fungal luciferin	Purtov, Yampolsky 2015	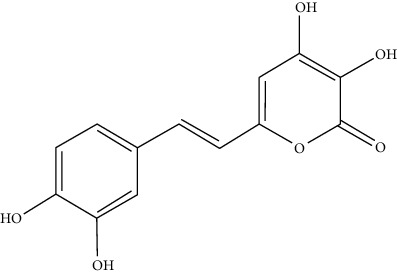

**Table 2:** Types of bioluminescence assays in research.

Type of application	Description	References
Reporter gene activity measuring	Red fluorescent protein, secretable *Gaussia* luciferase, stable and unstable nanoluciferase, and firefly luciferase have been used to study complex body fluids. Smad binding element (SBE) and NF*κ*B response element (NF*κ*B-RE) have been cloned to different reporter vectors	[47]
Single-reporter assays	To measure the potency of T-cell-dependent bispecific antibodies while capturing key aspects of its multistep mechanism of action, this assay has been used	[74]
Dual-reporter assays	To detect the alterations in the ratio of spliced and unspliced mRNA in mammalian cells, this assay has been used	[75]
Multicolor luciferase reporter assay	To investigate the visualization of the platform to investigate apoptosis and NF-*κ*B in human breast cancer cells	[76]
Real-time luciferase reporter assay	To detect the mammalian cell viability and the cytotoxicity of *β*-actin promoter and enhancer, this assay has been used	[77]

**Table 3:** Commercially available luciferase genes and their properties.

	Name of luciferin	Gene symbol	Organism	*I *_max_ (nm)	Mass (kDa)	
Nonsecretion type	Coelenterazine	Rluc	Sea pansy	480	36	[93]
	Firefly luciferin	CBGluc	Click beetle (Jamaica)	537	60	[94]
	Firefly luciferin	ELuc	Click beetle (Brazil)	538	61	[95]
	Firefly luciferin	SLG	Click beetle (Japan)	550	60	[96]
	Firefly luciferin	Luc(+), luc2	Firefly	562	61	[97]
	Firefly luciferin	SLO	Click beetle (Japan)	580	60	[94]
	Firefly luciferin	CBRluc	Click beetle (Jamaica)	613	60	[98]
	Firefly luciferin	SLR	Railroad-worm	630	61	[99]

Secretion type	Cypridinid luciferin	CLuc	Sea firefly (Ostracod)	465	61	[100]
	Coelenterazine	Gluc	Copepoda	480	20	[101]
	Coelenterazine	MetLuc	Copepoda	480	24	[102]

**Table 4:** Applications of bioluminescence in current research.

Type of application	Description	References
Reporter gene expression	In living subjects, for studying the magnitude, time variation, and location of reporter gene expression, an improved PET reporter gene tk (HSV1-sr39 thymidine kinase), and rl (*Renilla* luciferase, a bioluminescence optical reporter gene) are used	[112]

Assessing cell signaling pathways	NanoLuc luminescence has been documented as a tool for detecting both the overall levels of native target proteins and phosphorylation in unaltered cells through immunodetection	[113]

Bioluminescence-induced photo-uncaging of small molecules	The NanoLuc-Halotag chimera protein (H-Luc) induces BRET to a coumarin substrate, activating the coumarin's excited state. This activation, in turn, initiates hydrolysis, leading to the release of a caged target molecule	[114]

Bioluminescence-based photodynamic therapy (PDT)	Self-luminescent photodynamic therapy systems are used with potentials against viral infections	[115]

Bioluminescence imaging	*In vivo* glucose uptake evaluation	
	To noninvasive and real-time longitudinal imaging of glucose absorption in *in vivo* and *in vitro*, a novel bioluminescent glucose uptake probe (BiGluc) has been used	[116]
	As a technique to measure water pressure	
	*Pyrocystis lunula* has been used as a biological pressure sensor to measure the impulsive dynamic water pressure produced using weight-drop tests	[117]
	To study lung diseases	
	The expression of *Photinus* luciferase has been used to evaluate nuclear factor-*κ*B (NF-*κ*B) in rat models. By detecting the timing, distribution, and intensity, lung activities and inflammatory responses have been studied	[118]
	Cancer studies	
	Mouse xenograft model with bioluminescent Karpas-299 lymphoma cells has been used observed specific inhibition of lymphoma cell growth, and TAE684 exhibited potently	[119]
	In the exploration of T-cell priming events within secondary lymphoid tissues, luciferase-positive murine T regulatory cells (tregs) have been employed in an allogeneic bone marrow transplant model	[120]
	To study infectious diseases	
	Bioluminescent *Listeria monocytogenes* has been used to study pregnancy-related fetal infections	[121]
	Expression of firefly luciferase has been used to study infection by gamma-herpesviruses	[122]
	Bioluminescent *Neisseria meningitidis* has been used to monitor bacterial sepsis (sepsis disease, a meningitis-like disease, and a mild disease) in CD46 transgenic mice in real time	[123]
	Bacterial luxCDABE operon has been used to investigate parasite stage conversion and spore germination during infections	[124]
	To study cardiovascular diseases	
	To observe left anterior descending (LAD) artery ligation, murine embryonic stem (ES) cells with bioluminescence activity have been used	[125]
	To track surviving embryonic stem cells for up to 8 weeks, BLI is used	[121]
	To study central nervous system disorders	
	Transgenic mice have been used, and bioluminescent output from the main OBs (olfactory bulbs) of period1-FLuc has been tested	[126]
	To identify protein-protein interaction modulators	
	To quantify receptor ligand-binding events and their downstream signaling cascades, BRET and split luciferase assays are widely used	[127]
